# Honeybee adaptability to square comb foundation

**DOI:** 10.1038/s41598-026-45592-0

**Published:** 2026-03-28

**Authors:** Hiroyuki Shima, Maika M. Hayashi, Tadashi Kunieda, Makoto Honda, Shuji Sakai

**Affiliations:** 1 Department of Environmental Sciences, University of Yamanashi, 4-4-37, Takeda, Kofu, Yamanashi 400-8510 Japan; 2 Graduate School of Science and Technology, Nara Institute of Science and Technology, 8916-5 Takayama-cho, Ikoma, Nara 630-0192 Japan; 3 NPO Sapporo Honeybee Project, 12 Minami 4-jo, Higashi 2-chyome, Sapporo, Hokkaido 060-0054 Japan; 4 Hokkaido University of Education, 2-34-1 Midorigaoka, Iwamizawa, Hokkaido 068-8642 Japan

**Keywords:** Engineering, Materials science

## Abstract

In beekeeping, hexagonal comb foundation sheets made of beeswax are provided to bees beforehand to encourage them to build regular honeycomb cells. The practice leverages the bee instinct to construct cells of a specific size—a regular hexagon roughly the size of a bee head—when building their nests. However, it remains unclear how honeybees behave when presented with foundation sheets that deviate from their instinctively preferred shapes and sizes . In this study, we investigated the ability of bees to adapt to severe structural disturbances in cell geometry. To this end, foundation sheets composed of square indentations were provided to honeybees and their subsequent nest-building activities were observed periodically. Notably, honeybees keenly perceived the size and arrangement of the squares carved into the comb foundation and constructed different types of combs depending on the differences. The findings enhance our understanding of the mechanisms via which highly ordered hexagon honeycombs are constructed.

## Introduction

Honeybees (*Apis mellifera*) have been providing humans with honey and royal jelly for over 10,000 years. Relevant evidence was found in an ancient cave mural in the Cuevas de la Araña in eastern Spain, showing a woman reaching into a beehive to collect honey several thousand years ago^1^. In recent centuries, honeybees have continued to fascinate us, not only with their sweet preserves but also with their unique behaviours that stimulate human intellectual curiosity. For example, honeybees perform a waggle dance to communicate the location of a feeding area to nest mates^2–4^. In addition, they regulate the temperature inside a hive by flapping their wings in groups and circulating the air inside^5,6^. Additionally, they divide one honeycomb sheet into three concentric regions (a central nurturing region, a surrounding pollen region, and an outermost honey region) for different uses^7^. Furthermore, honeybees can perform numeric calculations^8,9^ and have a forensic eye that can distinguish between Monet and Picasso paintings^10^.

One of the most fascinating skills of honeybees is the ability to construct large orderly hexagonal comb structures. They construct hexagonal cell structures beaming with mathematical beauty^11–13^, in addition to being functional engineering marvels^14,15,^ by laying countless tiny tiles of beeswax secreted from glands in their abdomen^16,17^. Two major hypotheses have been proposed to explain how bees are able to accomplish such striking hexagonal lattices. The first hypothesis is that the beeswax hive, which is initially a cylindrical cluster (like a bundle of straws), is softened by bee body heat and then transformed into a hexagonal comb by the surface tension of the beeswax^18,19^. However, a counterargument asserts that beeswax does not soften sufficiently at the bee body temperature; therefore, mechanical shaping into hexagons does not occur^20,21^. According to the second hypothesis, an orderly hexagonal lattice is self-organised by a large number of bees acting according to simple behavioural rules^22^. That is, although there is no supervisor to oversee the whole, a regular honeycomb pattern is self-organised through the accumulation of individual actions that follow simple rules^23,24^. To date, which one of these two hypotheses is closer to the truth remains debatable. Nevertheless, time-resolved X-ray microscopy measurements^25^ have revealed that bees directly construct hexagonal cells, supporting the second hypothesis. Notably, hornets and paper wasps can form orderly hexagonal lattice structures using plant fibres, which are materials whose volumes remain unchanged even when heated^16,26,27^.

The idea proposed by the second hypothesis—that the behavioural principles of individual honeybees are the driving force behind the self-organisation of highly ordered hexagonal cells—is not surprising, considering honeybee nesting behaviour is sensitive to disturbance and adaptive^28–34^. For example, honeybees adapt skilfully to local deviations in the polygonal shapes of constituent honeycomb cells. It has been experimentally demonstrated that honeybees use non-hexagonal cells (e.g. pentagons, heptagons, and octagons) to merge two different honeycomb sheets extending from separate locations; in addition, they use non-hexagonal cells to spread different-sized hexagonal cells on a single honeycomb sheet with no gaps between cells^35–37^. These observations raise a simple question regarding the extent to which honeybees can adapt to forceful interruption of non-hexagonal cells: specifically, how can bees cope if geometric deviations from the regular hexagon are not restricted to a localised portion of the sheet, but are spread throughout the sheet? The latter corresponds to the case where the honeycomb sheet is filled with cells of congruent “squares” or “rectangles,” for instance.

The present study investigated the ability of bees to adapt to extremely strong honeycomb cell geometry disturbance. The bees were presented with a honeycomb foundation consisting of congruent square cells in the early stages of nest building, and how the bees piled beeswax onto them was observed. In the experiments, bees were forced to start their nest-building activity based a comb foundation with a square lattice. Hence, any attempt to alter the shape of a cell from a square to a hexagon was expected to encounter difficulties from a geometrical perspective. Observing how real honeybees overcome this strong geometric constraint to build their hives could offer insights into the mechanisms via which high-order hexagon honeycombs are constructed.

## Method

**Fig. 1:**
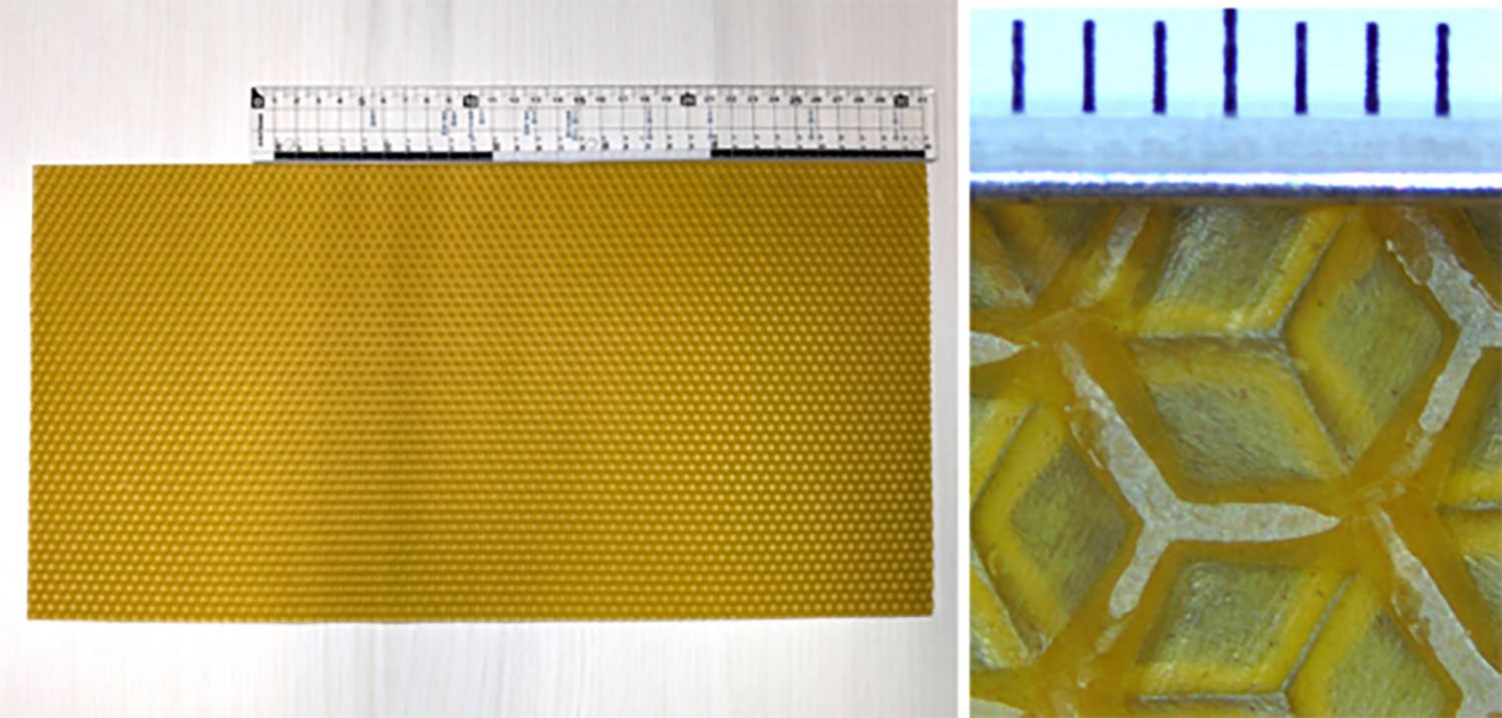
Left: photo of a commercially available comb foundation. Hexagonal ridges are carved on both the front and back sides. Right: enlarged photograph of the same foundation. The hexagons on the front and back are offset slightly relative to each other, so that the centre of the hexagon carved on the front aligns with the apex of the hexagon carved on the back. Each scale mark corresponds to 1 mm.

### Preparing comb foundations

**Fig. 2:**
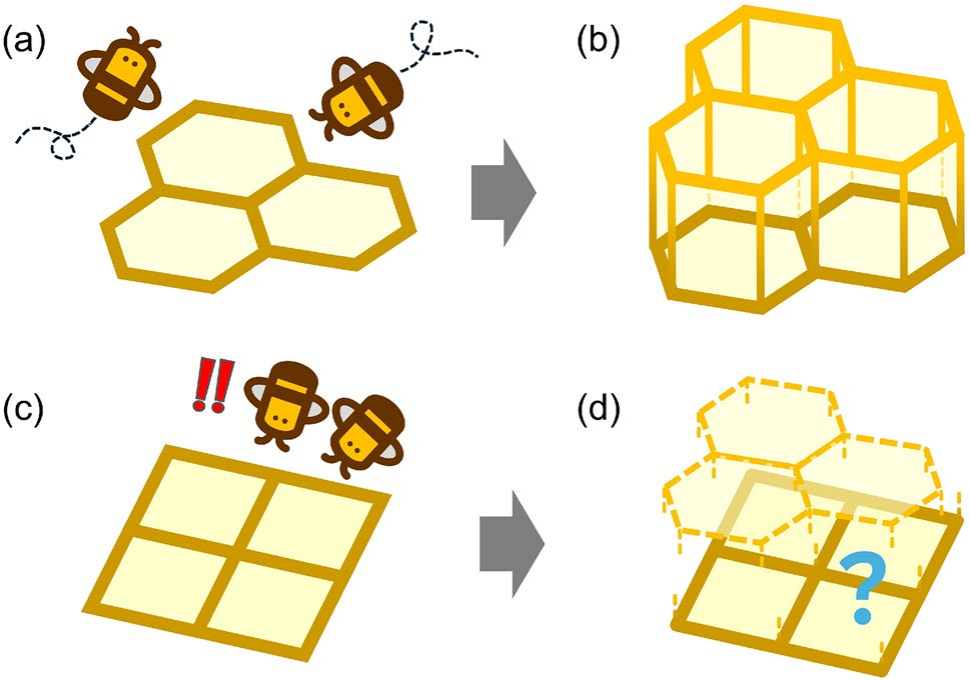
(**a**) When beekeepers provide frames with hexagonal ridges commonly used in beekeeping, the bees stack small tiles of beeswax on top of the frame walls to build them higher. (**b**) Consequently, highly ordered hexagonal prismatic honeycomb cells are completed rapidly. (**c**,**d**) If bees are provided with square-ridge foundations, what shape of cell walls would they construct? Resolving the question above is the objective of the present study.

In nature, wild honeybees build their hives from scratch in empty spaces such as in tree cavities or in house ceilings. Managed bees, conversely, are presented with a thin comb foundation made of artificial wax (see Fig. 1) to facilitate building of seamless hexagonal columnar spaces for larva and pupa care and honey storage over a relatively short period, as illustrated in Fig. 2a,b. The front and back faces of the hexagonal comb foundation are moulded into small regular hexagonal ridges. The length of one side of the hexagon is approximately 3 mm, and the width and height of the ridge are both approximately 1 mm. The values mimic the size of a hexagonal hive cell made by wild bees. The base of a foundation hexagon is essentially flat, whereas wild honeybees form regular bumps and hollows at the bottom of their nests^38^.

In practice, a single flat comb foundation is fixed inside a rectangular wooden frame. Several wooden frames are placed parallel to each other and hung in a hive box where the honeybees are kept. Over time, the bees pile beeswax on top of the pre-carved hexagonal ridges to increase the heights of walls of individual cells. Recent studies have reported that honeybee nest-building abilities vary across species^39^, and in Japanese beekeeping, the Italian honeybee, which has a high nest-building ability, is primarily used.

**Fig. 3:**
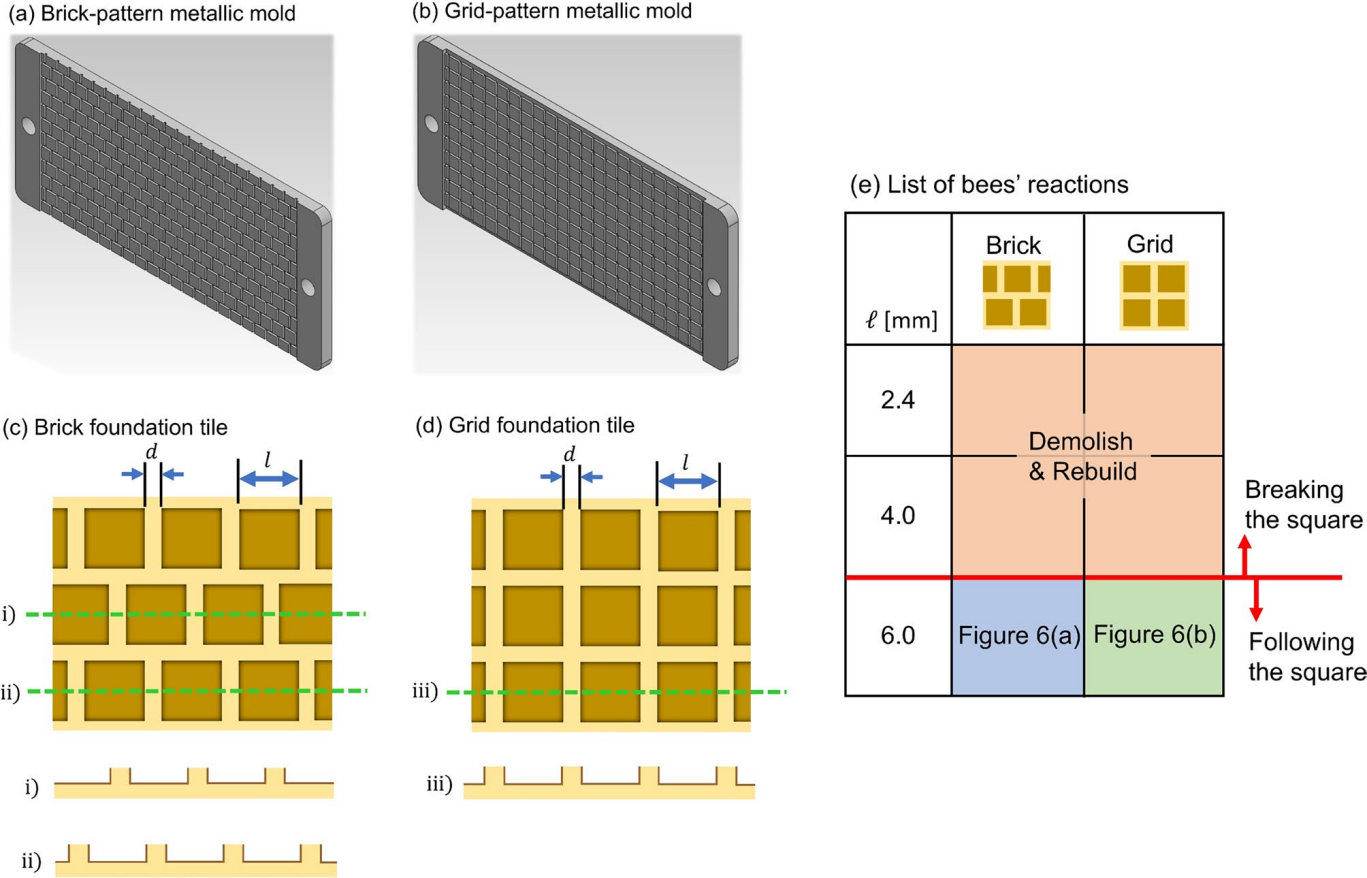
(**a**,**b**) Images of metal moulds used to create square-ridge comb foundation made by beeswax. (**a**) Brick-like pattern mould; (**b**) Grid-like pattern mould. (**c**,**d**) Diagrams of beeswax-made tiles with periodically arranged square depressions. A grid pattern (**c**) or brick pattern (**d**) of square depressions (each side length $\ell$) was carved onto the surface of a plate made by cooling and solidifying melted beeswax, with a constant spatial period (gap *d* between adjacent squares). The cross-section when the comb foundation was cut at the position indicated by the green dotted line is shown at the bottom of the figure. (**e**) Summary of honeybees’ reactions caused by differences in the square arrangement and edge length $\ell$. When $\ell \le 4.0$ mm, the bees destroyed the square ridges and rebuilt them into standard hexagonal cells. When $\ell =6.0$ mm, the bees deposited beeswax on top of the square ridges, while the shape of the finished cells varied depending on the square arrangement. Refer to the main text for details.

In the present study, custom-fabricated square-lattice comb foundations were used instead of the typical hexagonal lattice comb foundation (see Fig. 2c,d). First, multiple moulds with square grooves were prepared; two types of moulds with different groove shapes are illustrated in Fig. 3. The length of one side of the squares engraved on the mould surface, denoted by $\ell$ in Fig. 3c,d, was set to three different values: 2.4, 4.0, and 6.0 mm. Groove width (*d*) and depth were fixed at 1.0 mm for all the prepared moulds. The square arrangement was set in the following two patterns. The first is a brick-like pattern (see Fig. 3c), where three straight grooves intersect at the square vertices to form T-junctions. The second is a grid-like pattern (Fig. 3d), where four grooves intersect at the square vertices in a cross shape. To summarize briefly, this study focused on two factors—square size (three values: 2.4, 4.0, and 6.0 mm) and arrangement pattern (two types: brick and grid)—and created a total of six types ($3\times 2$) of moulds for producing beeswax tiles; see Fig. 3e.

After the moulds were completed, liquid wax made by melting a commercially available hexagonal foundation was poured into the gap between the paired moulds with the same type and left to cool and solidify. Using this method, approximately 20 beeswax tiles were created for each fixed $\ell$ and fixed square arrangement pattern. The dimensions and cross-sectional views of the prepared beeswax tiles with two different patterns are shown in Fig. 3c,d. By arranging and securing the tiles inside a wooden frame, square-ridge comb foundations with sizes similar to those of commercially available hexagonal ridge foundations were completed. Subsequently, how honeybees built up the cell walls based on the square foundation was observed.

### Beekeeping conditions and observation methods

Beekeeping and observation of comb construction process were conducted on the rooftop of a seven-story building in the centre of Sapporo city, Japan (43$^\circ$ 03’30.1”N, 141$^\circ$ 20’58.7”E). The breeding periods were April-September 2023, and April-September 2024. During this period, full-scale observations were conducted from June through September each year, when nectar secretion was sufficiently abundant. On the observations, the hive box was opened every few days to check the growth of the cell walls and bee activity. The specific dates on which the interior of the hive was visually inspected are listed in Supplementary Information.

At the start of the beekeeping period, there were approximately 3,000 bees (one colony) in one hive box, and four frames (including square foundation) were installed inside the hive. Over the course of the several-month beekeeping period, as the bee population grew, the number of hive boxes and frames was increased (reaching a maximum of two hives, with nine frames per hive).

When inserting square ridge comb foundations into the hive box, they were positioned near the centre of the hive box, with multiple hexagon-ridge comb foundations arranged parallel to each other in front and behind. This is because honeybee comb-building activity is generally most active near the centre of the hive box. Between facing beehive frames, there is a gap approximately the width of a single bee, which allows the bees to move freely between different beehive frames. Except for the unusual shapes of ridges carved into the foundation, beekeeping was conducted as usual.

It would be worth mentioning the preparations needed before observing nest-building behavior (i.e., from April to May each year). First, in April, we placed sugar water and pollen substitutes inside the hives as supplementary food to protect the colonies—which had become malnourished after enduring the winter and early spring—from starvation. Then, once May arrived and we confirmed that the bees had begun collecting nectar from nectar sources, we removed all supplementary food from the hive and cleaned all the frames using a honey extractor. This cleaning process was performed to ensure that the sugar water and other feed did not mix with the honey the bees were gathering into the cells. After completing these tasks, we allowed the bees to begin full-scale honey collection activities starting in June.

## Results and discussion

### 2.4 and 4.0-mm cases: nest building “ignoring” square ridges

**Fig. 4:**
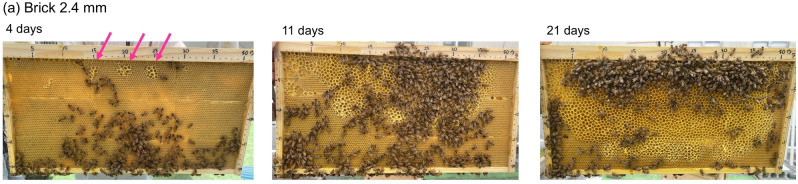
Honeycomb construction process when 2.4-mm-sided square depressions are carved in a brick-like pattern: (**a**) 4 days after installing the square foundation; (**b**) 11 days later; (**c**) 21 days later. The honeybees broke down the square walls carved into the initial comb foundations and built hexagonal walls of a preferred size.

Figure 4 shows the results of the periodic observations conducted by temporarily removing only the square-lattice comb frames from the hive. Photographs illustrate the state of the nest, constructed gradually starting from the brick-pattern foundation with a square side length of $\ell =$2.4 mm, at (a) 4 days, (b) 11 days, and (c) 21 days after construction began. In this case, the size of the cells initiated at the location indicated by the pink arrows is clearly larger (diameter of approximately 6.0 mm) than the size of the square depressions placed on the foundation tiles (2.4 mm per side). That is, the honeybees created cells with preferred sizes, disregarding the square ridges carved into the foundation tiles.

Upon close examination of the bottom surface of the cell, the square ridges that were present in the initial state disappeared. This suggests that the honeybees removed the square walls, levelled the surface of the foundation locally, and likely reused the scraped-off beeswax to construct hexagonal walls. This forceful nest-building continued thereafter, ultimately resulting in the construction of a nest with high hexagonal symmetry (completely unrelated to the originally carved square ridges). Notably, such a forceful nest-building process was observed in both the brick-like and grid-like patterns for both 2.4- and 4.0-mm square cases.

### 6.0-mm cases: nest building “along” square ridges

As mentioned above, honeybees provided with a 2.4- or 4.0-mm square pattern foundation constructed hexagonal cells disregarding the precarved square walls, regardless of whether the pattern was brick-shaped or grid-shaped. In contrast, when provided with 6.0-mm square patterns, the honeybees integrated the square ridges carved into the initial foundation.

**Fig. 5:**
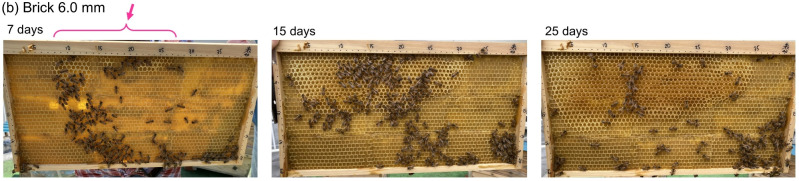
Honeycomb construction process results when 6-mm side square depressions were carved in a brick-like pattern: (**a**) 7 days after installing the square foundation; (**b**) 15 days later; (**c**) 25 days later. The honeybees layered beeswax in nearly the same position as the square walls carved into the initial comb foundation, altering the orientation and thickness of the walls gradually to complete hexagonal walls of a preferred size.

Figure 5 shows the nest-building process starting from a brick-pattern foundation with a square length of 6.0 mm, at (a) 7 days, (b) 15 days, and (c) 25 days after construction began. In Fig. 5a, thick-walled hexagonal cells begin to form at the top edge of the nestboard, as indicated by the pink arrow. The size of the hexagonal cells is nearly identical to that of the square cells that were initially carved into the comb. Subsequently, the honeybees expanded the thick-walled hexagonal cells across the entire comb, as shown in Fig. 5b,c. The comb construction rate was nearly identical to that observed for a typical hexagonal foundation installed in the same hive box. That is, even when provided with a 6.0-mm square “brick-pattern” foundation, the honeybees constructed comb with a high degree of hexagonal symmetry, nearly identical to the results when provided with the standard hexagonal foundation (with each side of the hexagon being ca. 3.0 mm). Similar construction behaviour was observed when using a 6.0-mm square “grid-pattern” foundation. However, as described subsequently, the arrangement of cells in the completed comb was entirely different from that of the brick-like pattern.

### Pattern effect difference for 6.0 mm cases: brick versus grid

**Fig. 6:**
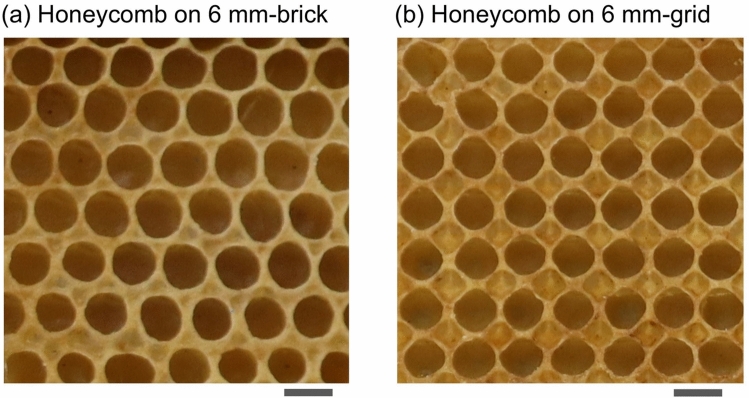
Close-up photograph of a mature honeycomb constructed from square foundation cells measuring 6 mm per side. (**a**) Brick-pattern foundation cells; (**b**) grid-pattern foundation cells. Scale bar: 6 mm.

We have demonstrated in the previous paragraph that, when provided with a foundation featuring 6.0-mm square ridges, honeybees built their combs by stacking beeswax onto the ridges, regardless of whether the square arrangement was brick- or grid-like. An interesting finding was that the final arrangement of cells in the completed comb differed visually between the brick-like and grid-like patterns. Figure 6 shows how the comb structures built by honeybees, given an initial 6.0-mm square foundation, differ between brick-like and grid-like patterns. As is apparent from the photographs, the brick-like ridge pattern induced a hexagonally symmetrical arrangement of cells. In contrast, the arrangement of cells formed on the grid pattern lost hexagonal symmetry and exhibited four-fold rotational symmetry, similar to a square.

Another key finding is the crucial difference between the cell arrangement formed on a 6.0-mm brick-like pattern and that formed on a commercially available hexagonal comb foundation, while both arrangements exhibit hexagonal symmetry. The difference lies in the fact that in the former arrangement, adjacent cells do not share any hexagonal edges. As shown clearly in Fig. 6a, the wall shared between two adjacent cells is only a small portion (or nearly a single point) of the cell circumference. Thus, the honeycombs built by bees on top of the brick-like pattern contained numerous dead spaces that were unusable for honey storage or brood rearing. This dense packing of the rounded cells separated by dead spaces closely resembles the structure of wet foam (a state in which liquid-rich spherical bubbles are packed densely)^40^.

Even more intriguing is the arrangement of the cells shown in Fig. 6b—specifically, the structure built by the bees atop the 6.0-mm grid pattern. In such a case, the centre of each rounded cell aligns with the centre of the square carved into a grid-shaped comb foundation. Furthermore, small bowl-shaped membranes of beeswax, resembling inverted pyramids, are formed at the vertices of the square carved into the comb foundation. This distinctive cell structure, with the hexagonal symmetry lost completely, represents a compromise between adaptability to the unusual cell arrangement originally carved into the grid-shaped comb foundation and inability to construct square cell walls without gaps over the entire sheet.

### Geometric reason behind the self-organisation of the three different cell structures

Our observations revealed that the cell structures built by honeybees on square foundation sheets could be classified broadly into the following three types. The first type consists of hexagonal cells constructed to the size preferred by honeybees, disregarding the squares of the foundation. The structure is built when the engraved square size is either 2.4 or 4.0 mm, independent of the square arrangement pattern. The second type is an array of rounded cells exhibiting hexagonal symmetry built on 6.0-mm-sided brick-like patterns. In the third type, rounded cells are arranged in a square grid pattern, which is constructed when a 6.0-mm-sided grid pattern is used.

Why do honeybees disregard the provided ridges entirely and rebuild honeycombs from square to hexagonal when the square is small? Conversely, why do honeybees utilise the provided ridges and stack beeswax on top of them when the square size is large? The outcomes could be explained by comparing the hexagonal shape and size preferred by honeybees with the square shape and size carved into the foundation.

**Fig. 7:**
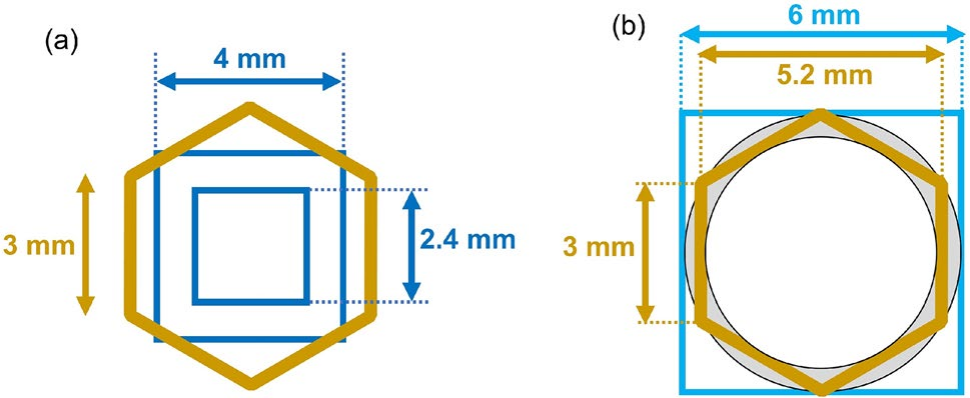
(**a**) Comparison of dimensions between the regular hexagonal depressions (3 mm per side) carved into commercially available foundation and the square depressions (side lengths 2.4 and 4.0 mm respectively) created for the experiment. (**b**) Comparison between the hexagon (3 mm per side) and the square with 6.0 mm per side. The distance between any two opposite sides of the hexagon is approximately 5.2 mm, which is slightly smaller than that of the square.

Figure 7a shows a dimensional comparison between the hexagons preferred by honeybees (with a side length of 3.0 mm) and the small squares used in this experiment (with side lengths of 2.4 or 4.0 mm). In this case, the squares are clearly smaller than the hexagon (slightly larger than the honeybee head). That is, the distance between opposite sides is too narrow, making it difficult for honeybees to use their body to stack beeswax onto the ridge. This could be the reason why honeybees remove such disruptive square ridges and rebuild their preferred hexagonal walls on a locally levelled surface. Notably, similar behaviour of destroying pre-carved ridges has been observed when honeybees are presented with hexagonal ridge comb foundations that are smaller than the standard size^34^. That is, honeybees possess such proactive creativity that when presented with comb foundations too small to be useful, they dismantle the foundations and construct new cells, regardless of their original shape.

**Fig. 8:**
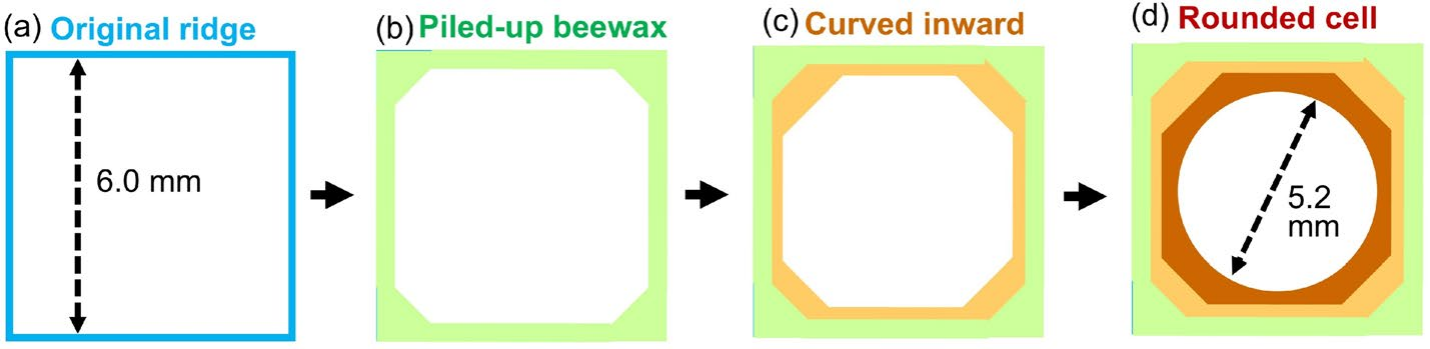
Schematic diagram of the process of accumulating beeswax tiles onto a square ridge. (**a**) A square with a side length of 6.0 mm carved into the foundation. (**b**) Beeswax piled up onto the ridge in the early stages of comb construction. (**c**) Over time, when adding wax near the square vertices, the walls begin to curve inward, drawing closer to the square centre. (**d**) Ultimately, a rounded cell of the size preferred by bees is completed.

Even more notable, when the side length of the square carved into the foundation is 6.0 mm (see Fig. 7b), the honeybees exhibit completely distinct responses. In the case above, the distance between opposite sides of the carved square is close to the distance between opposite sides of the hexagon preferred by honeybees (approximately 5.2 mm). It is inferred that the honeybees compensated for the slight inconvenience by stacking beeswax atop the square protrusions to build higher walls (see Fig. 8a,b). However, during construction, instead of extending the square walls perpendicular to the sheet, the bees curved the stacked beeswax inwards towards the centre of the square (Fig. 8c). This allows the bees to bring the spacing between opposing walls closer to their preferred interval (approximately 5.2 mm), whereas the wall thickness separating the adjacent cell spaces increases. In particular, the ridge near the square vertices was far from the centre of the square, deviating largely from the wall spacing preferred by honeybees. Therefore, when attaching beeswax to the ridge near the square vertices, the bees exhibited a pronounced tendency to stack it with a curvature toward the centre of the square. Consequently, the cell walls of the resulting honeycomb are interpreted as being slightly smaller than the original square indentations and having a rounded shape (Fig. 8d). Based on this simple behavioural principle, as honeybees stack beeswax onto the wall, the arrangements of rounded cells are completed atop the 6.0-mm brick- and 6.0-mm grid patterns.

### Open questions remained

Below, we present several persisting knowledge gaps and potential avenues for future research.

Our observations revealed that honeybees employ two distinct types of nest-building behaviour depending on the size of the square foundation provided: either “breaking the square” or “following the square.” The threshold value for the length of one side of the square, which marks this boundary, should lie between 4.0 and 6.0 mm. Consequently, what is the specific threshold in millimetres? In addition, which part of the honeybee body determines the threshold?

To resolve the question, the sizes of the square foundation blocks used in the present study need to be increased or decreased systematically and the responses of the bees to each foundation block should be observed. The results would provide key insights on the most fundamental rule (length standard) governing the bee behaviour in the course of comb construction. It is also noteworthy that in the experiment we have conducted, only a single colony belonging to a single species was used. Therefore, we cannot rule out the possibility that the threshold values would differ if the species or colony were different. Similarly, the same possibility exists if the experiment were conducted at a different field conditions (i.e., season, location, beekeeping methods, etc).

Exploring the influence of square-ridge comb foundations on the size of the cells and the spatial order of their arrangement would also be insightful. Our rough estimation revealed that the area of the rounded cells formed from the square foundation was slightly larger than that formed from the standard hexagonal cells. This is consistent with our observation that cells built on a square foundation were more frequently used to raise male honeybees (slightly larger than females). Therefore, a question of how the size of the square is related to the size of the cell built from the square arises. Thorough and high-precision image analysis may reveal a correlation with cell area or cell arrangement, which may be quantified based on the bond orientation order parameter.

As somewhat unconventional ideas, experiments using comb foundations with pseudo-five-fold rotational symmetry, such as Penrose tiling^41^, or experiments using comb foundations that fill the entire plane with the same shape as Stone-Wales defects (pairs of pentagons and heptagons) in two-dimensional carbon bonds^42^ are also notable. Honeybees can effectively utilise pentagonal or heptagonal cells to complete their honeycombs. Therefore, if the entire foundation initially provided is based on pentagonal or heptagonal cells, rather than simply ignoring them, they may employ a compromise or proactive strategy to utilise the non-hexagonal foundation effectively.

As a final note, we emphasize that observation of responses to altered early-stage comb foundation shapes could facilitate the elucidation of the rules governing honeybee comb-building behaviour. Generally, honeybee nest-building behavior has been often considered rigidly constrained by innate instinctive patterns, lacking flexibility. However, a series of past experimental studies revealed that honeybees acutely perceive local structural irregularities and actively work to overcome them, ultimately completing high-quality cell networks^28–37^. The findings of this study should be significant in that they further deepen this understanding. From another perspective, research based on observation, such as that conducted in this experiment, is also useful for research based on simulation. In fact, simulation-based methods have been revealed to be one useful method for resolving the honeybee problem, incorporating behavioural principles that individual honeybees might apply. To validate the assumptions (individual bee behavioural rules) incorporated into such programs, an effective approach is to observe bee nest-building behaviour when provided with comb foundations with different geometric characteristics, as in this and other studies. Integrating such observations into simulations would facilitate the creation of more realistic nest-building simulations.

## Summary

The aim of the present study was to elucidate the rules governing individual honeybee nest-building behaviour by providing bees with comb foundation sheets with square ridges and observing their subsequent nest-building activities. The results showed that honeybees distinguished the sizes of the squares and adjusted their construction accordingly. Specifically, when the side length of the square depression was smaller than that of a standard hexagonal cell (with a diagonal length of approximately 6.0 mm), the honeybee dismantled the existing square ridges and built a hexagonal cell from a locally flat site. Conversely, when the side length of a square depression was nearly the same as that of a standard hexagonal cell, the honeybee utilized the square ridge to construct the cell. Notably, in the latter case, the arrangement of cells in the mature comb differed (hexagonal lattice or square lattice) depending on the configuration of the square ridges (brick or grid pattern). Our findings suggest that when cell walls are extended by the accumulation of beeswax tiles, honeybees prioritise the spacing between opposite sides of the polygons carved into the foundation.

## Supplementary Information

Supplementary Information.

## Data Availability

The datasets used in the current study will be available from the corresponding author on request.
