# Supplementary material for: Honeybee adaptability to square comb foundation
Source: Sci Rep. 2026 Mar 28;16:10816. doi: 10.1038/s41598-026-45592-0 (PMC13039314; doi:10.1038/s41598-026-45592-0)
Supplement: Supplementary file 1 — Supplementary Information. [file 41598_2026_45592_MOESM1_ESM.docx]

Supplementary Information

[ H. Shima et al., “Honeybee Adaptability to Square Comb Foundation”, Scientific Reports (2026)]

The following is a list of the dates on which visual inspections of the hive interior were conducted (as well as the periods during which each type of square foundation with different dimensions was placed inside the hive).

i) 2.4 mm brick & grid:

August 12, 15, 19, 26, 29 / September 2, 5, 9, 12, 16, 19, 23, 26/ in 2024.

ii) 4.0 mm brick & grid:

July 24 / August 1, 7, 10, 14, 17, 21, 24, 28, 31 / September 4, 7, 11, 21 / in 2023.

iii) 6.0 mm brick & grid:

June 23, 27 / July 4, 8, 11, 15, 18, 22, 25 / August 1, 5, 8 / in 2024.
